# Microbial community composition shapes enzyme patterns in topsoil and subsoil horizons along a latitudinal transect in Western Siberia

**DOI:** 10.1016/j.soilbio.2015.01.016

**Published:** 2015-04

**Authors:** Jörg Schnecker, Birgit Wild, Mounir Takriti, Ricardo J. Eloy Alves, Norman Gentsch, Antje Gittel, Angelika Hofer, Karoline Klaus, Anna Knoltsch, Nikolay Lashchinskiy, Robert Mikutta, Andreas Richter

**Affiliations:** a University of Vienna, Department of Microbiology and Ecosystem Science, Division of Terrestrial Ecosystem Research, Vienna, Austria; b Austrian Polar Research Institute, Vienna, Austria; c University of Vienna, Department of Ecogenomics and Systems Biology, Division of Archaea Biology and Ecogenomics, Vienna, Austria; d Leibniz Universität Hannover, Institut für Bodenkunde, Hannover, Germany; e Aarhus University, Center for Geomicrobiology, Department of Bioscience, Aarhus, Denmark; f University of Bergen, Centre for Geobiology, Bergen, Norway; g Central Siberian Botanical Garden, Siberian Branch of Russian Academy of Sciences, Novosibirsk, Russia

**Keywords:** Extracellular enzymes, PLFA, Tundra, Boreal forests, Steppe, Permafrost

## Abstract

Soil horizons below 30 cm depth contain about 60% of the organic carbon stored in soils. Although insight into the physical and chemical stabilization of soil organic matter (SOM) and into microbial community composition in these horizons is being gained, information on microbial functions of subsoil microbial communities and on associated microbially-mediated processes remains sparse. To identify possible controls on enzyme patterns, we correlated enzyme patterns with biotic and abiotic soil parameters, as well as with microbial community composition, estimated using phospholipid fatty acid profiles. Enzyme patterns (i.e. distance-matrixes calculated from these enzyme activities) were calculated from the activities of six extracellular enzymes (cellobiohydrolase, leucine-amino-peptidase, N-acetylglucosaminidase, chitotriosidase, phosphatase and phenoloxidase), which had been measured in soil samples from organic topsoil horizons, mineral topsoil horizons, and mineral subsoil horizons from seven ecosystems along a 1500 km latitudinal transect in Western Siberia. We found that hydrolytic enzyme activities decreased rapidly with depth, whereas oxidative enzyme activities in mineral horizons were as high as, or higher than in organic topsoil horizons. Enzyme patterns varied more strongly between ecosystems in mineral subsoil horizons than in organic topsoils. The enzyme patterns in topsoil horizons were correlated with SOM content (i.e., C and N content) and microbial community composition. In contrast, the enzyme patterns in mineral subsoil horizons were related to water content, soil pH and microbial community composition. The lack of correlation between enzyme patterns and SOM quantity in the mineral subsoils suggests that SOM chemistry, spatial separation or physical stabilization of SOM rather than SOM content might determine substrate availability for enzymatic breakdown. The correlation of microbial community composition and enzyme patterns in all horizons, suggests that microbial community composition shapes enzyme patterns and might act as a modifier for the usual dependency of decomposition rates on SOM content or C/N ratios.

## Highlights

• Enzyme patterns differed more strongly between horizons than between ecosystems.
• Enzyme patterns in subsoils vary more strongly than enzyme patterns in topsoils.
• Enzyme patterns in topsoil are related to SOM content and mic. community composition.
• Enzyme patterns in mineral subsoil were not related to SOM content or SOM C/N.

## 1 Introduction

Extracellular enzymes break down soil organic matter (SOM) at every depth of the soil profile. Nonetheless most studies on enzyme activities focused on topsoil horizons in the upper 20 cm of the soil profile (e.g. Sinsabaugh et al., 2008; Wallenstein et al., 2009; Kaiser et al., 2010) although up to 60% of the carbon stored in soils are located below 30 cm (Jobbágy and Jackson, 2000). These subsoil horizons differ from well studied topsoil horizons in a number of physical and chemical conditions that might influence enzyme activities and decomposition in general (Rumpel and Kögel-Knabner, 2011): Temperature decreases from topsoils to subsoils whereas soil moisture increases with depth, either improving conditions for decomposition in arid systems (Rovira and Vallejo, 2002), or impairing them in systems where water logging occurs and O_2_ availability is low (Kleber, 2010; Davidson et al., 2012). Soil pH, one of the factors often associated with enzyme activities (Sinsabaugh et al., 2008), also changes with depth (Eilers et al., 2012). In addition to these direct influences on enzyme activities, the availability of substrate for enzymatic breakdown decreases with depth. First, SOM is less abundant in subsoils, which leads to a high probability of a spatial disconnection of enzyme and substrate (Holden and Fierer, 2005). Second, a high proportion of SOM in subsoils is bound to minerals, stabilized by metal ions, or occluded in aggregates and therefore access for microorganism is limited (von Lützow et al., 2006). In addition to physical hurdles for decomposition, SOM in subsoils is chemically different from topsoil SOM. While the main proportion of SOM in topsoils is plant derived material, SOM in subsoils is microbially transformed (Wallander et al., 2003). During this microbial transformation of SOM, carbon is lost, mainly as CO_2_, whereas most of the nitrogen (N) is recycled and remains in the system, resulting in lower C/N ratios of subsoil SOM (Rumpel and Kögel-Knabner, 2011).

To fulfill the microbial demand for energy and nutrients, microorganisms need to adapt to the chemical composition of SOM and to the C/N ratio of the available substrate by adjusting their enzyme production (Sinsabaugh et al., 2008). Changes in enzyme production might either be physiological (Stone et al., 2014) or they might result from a shift in microbial community composition (Kaiser et al., 2014). Although the influence of microbial community composition on major microbial processes, such as C mineralization and N mineralization, has been recently challenged (Colman and Schimel, 2013), its influence on enzyme activities has been demonstrated repeatedly (e.g. Strickland et al., 2009; McGuire and Treseder, 2010; Schnecker et al., 2014). Microbial community composition, as another potential control on enzyme activities, has already been shown to change more strongly with soil depth, within ecosystems, than between topsoils of different ecosystems (Eilers et al., 2012; Gittel et al., 2014).

Relations of enzyme activities to key factors such as pH, moisture, SOM content (Keeler et al., 2009), chemical composition of SOM (Grandy et al., 2009; Sinsabaugh and Follstad Shah, 2010), and microbial community composition (Waldrop and Firestone, 2006; Talbot et al., 2013) are well established in topsoil horizons. Whether enzyme activities in the subsoils are related to these key factors is still largely unknown since few studies have addressed changes of enzyme activities and their potential controls with soil depth so far (e.g.: Brockett et al., 2012; Kramer et al., 2013; Turner et al., 2014; Schnecker et al., 2014; Stone et al., 2014).

In this study we investigated enzyme patterns in different soil horizons, including mineral subsoils, from a wide range of ecosystems to identify potential drivers for these enzyme patterns. We measured potential activities of six extracellular enzymes in organic topsoil horizons, mineral topsoil horizons and mineral subsoil horizons in seven ecosystems along a 1500 km-long north–south transect in Western Siberia. In addition to enzyme activities, we analyzed microbial community composition (using phospholipid fatty acid analysis) as well as abiotic soil parameters and related these factors to the enzyme patterns.

We hypothesized: (1) enzyme patterns in topsoil and subsoil horizons are both related to the same key parameters, such as SOM content, pH and microbial community composition. Microbial community composition has been shown to differ more strongly between topsoils and subsoils than between topsoils of different ecosystems (Meyer et al., 2006; Eilers et al., 2012). Since enzyme activities and enzyme patterns are often related to microbial community composition; (2) enzyme activities and enzyme patterns change with depth and differ more strongly between horizons than between ecosystems. The ecosystems along the transect showed large differences in vegetation and presumably in the chemical composition of litter entering the soil; (3) enzyme patterns would be more variable and show greater differences between ecosystems in the topsoil horizons, where the main constituents of SOM are plant-derived, than in mineral subsoil horizons.

## 2 Material and methods

### 2.1 Sampling sites

Soil samples were taken from seven ecosystems along a 1500 km latitudinal transect in Western Siberia, in August and September 2012. The ecosystems included tundra, northern taiga, middle taiga, and southern taiga, forest steppe (one forest site and one meadow site), and steppe. All soils were sampled from the active layer in an unfrozen state. Basic soil and climate parameters are provided in Table 1 and Table S1. Climate data are derived from Stolbovoi and McCallum (2002), soil classification follows the World Reference Base for Soil Resources (IUSS Working Group WRB, 2006).

At all sites, we sampled the three dominant soil horizons of five replicate soil pits. We categorized the three horizon types as organic topsoil horizon (uppermost horizon, O), mineral topsoil horizon (second horizon, A), and mineral subsoil horizon (third horizon, M). We removed living plant roots from the samples and sieved them to <2 mm. We did this for samples from all sites, except for the tundra, where samples were manually homogenized because they were too moist for sieving. Before further analyses, soil water content was adjusted to a minimum of 60% for organic topsoils (except steppe), to 15% for mineral topsoils and steppe organic topsoils, and to 10% for mineral subsoils, respectively.

### 2.2 Soil parameters

Soil pH was determined in 1 M KCl extracts. Samples for determination of organic C, total N content, and δ^13^C were dried at 60 °C and ground with a ball mill. Ground samples were analyzed with EA-IRMS (CE Instrument EA 1110 elemental analyzer, coupled to a Finnigan MAT DeltaPlus IRMS with a Finnigan MAT ConFlo II Interface, Thermo Fisher Scientific, Waltham, MA, USA). Mineral topsoils and subsoils at both forest steppe sites, as well as all horizons of the steppe site, contained traces of carbonate. Carbonate was removed from these samples by acidification with HCl before EA-IRMS analysis. Water holding capacity (WHC) was determined as the amount of water that remained in saturated soil, from which water could be lost by drainage but not by evaporation after two days (Reynolds and Topp, 2007).

Microbial C and N were estimated using chloroform-fumigation–extraction (Kaiser et al., 2010 modified after Brookes et al., 1985): Soil samples, fumigated with chloroform, as well as unfumigated samples were extracted with 0.5 M K_2_SO_4_. Dissolved organic C and total dissolved N were determined in both sets of extracts with a DOC/TN analyzer (Shimadzu TOC-VCPH/CPN/TNM-1, Vienna, Austria). Microbial C and N were calculated as the difference between fumigated and non-fumigated samples, without correction for extraction efficiency. C/N ratios of SOM and microbial biomass were calculated on a mass basis.

### 2.3 Potential extracellular enzyme activities

We measured potential enzyme activities fluorimetrically and photometrically using a microplate assay (Kaiser et al., 2010). For the fluorimetric assay, we used MUF (4-methylumbelliferyl) labeled substrates: β-d-cellobioside for cellobiohydrolase (CBH), triacetylchitotrioside for chititriosidase (CHT), N-acetyl-β-d-glucosaminide for N-acetyl-glucosaminidase (NAG) and phosphate for phosphatase (PHO). l-leucine-7-amido-4-methyl coumarin was used as substrate for leucine-amino-peptidase (LAP). Phenoloxidase (POX) activities were measured using L-3,4-dihydroxyphenylalanine (DOPA) as substrate in a photometric assay. Assays for CBH, CHT, NAG, PHO and LAP were incubated for 140 min at room temperature in a sodium acetate buffer (pH 5.5) and activity was measured fluorimetrically (excitation 365 nm and emission 450 nm). Plates for POX activity were measured photometrically (absorbance 450 nm) at the beginning and after incubation for 20 h at room temperature. POX activity was than calculated as the increase in color during the incubation time.

### 2.4 Phospholipid fatty acid (PLFA) analysis

Extraction and measurement of PLFAs followed the procedure described by Frostegård et al. (1991) with the modifications by Kaiser et al. (2010). PLFAs were extracted from 1 g soil with chloroform/methanol/citric acid buffer and purified on silica columns (LC-Si SPE, Supleco, Bellefonte, PA, USA) using chloroform, acetone, and methanol. After addition of the internal standard (methyl-nonadecanoate), PLFAs were converted to fatty acid methyl esters (FAMEs) by alkaline methanolysis. Samples were analyzed on a Thermo Trace GC with FID detection (Thermo Fisher Scientific, Waltham, MA, USA), using a DB-23 column (Agilent, Vienna, Austria). FAMEs were identified using qualitative standard mixes (37 Components FAME Mix and Bacterial Acid Methyl Esters CP Mix, Supelco) and quantified using the internal standard. We categorized the fatty acids according to Kaiser et al. (2010). The markers 18:1ω9, 18:2ω6,9, and 18:3ω3,6,9 were used as markers for fungi; i15:0, a15:0, i16:0, i17:0, a17:0, cy17:0 (9/10), cy19:0 (9/10), 16:1ω7, 16:1ω9, 18:1ω7, 15:0, and 17:0 as bacterial markers. We used the above mentioned markers together with 14:0, i14:0, 16:0, 18:0, 20:0, 22:0, 16:1ω11, and 19:1ω8 for the calculation of total PLFA content (Schnecker et al., 2012).

### 2.5 Statistics

We calculated enzyme patterns to identify differences between horizons and between sites. To account for the different methods of measuring enzyme activities and the inherent differences in enzyme activities of different horizons, the individual enzyme activities per gram dry soil were log transformed and standardized by calculating the proportion of each enzyme to the sum of all enzymes. With these values, we calculated Euclidean distance matrixes. We used these matrixes to create Nonmetric Multidimensional Scaling (NMDS) plots. To identify differences between sites and horizons we used Permutational Multivariate Analysis of Variance Using Distance Matrices (ADONIS). This analysis is implemented in the R-package vegan (Oksanen et al., 2013). Additionally we used Mantel tests based on Spearman correlations of the calculated enzyme distance matrices with soil parameters and with microbial community composition (represented as a distance matrix based on relative abundances of individual PLFA biomarkers). We performed these analyses for the whole data set, as well as for data sets of the three horizon classes individually.

To evaluate whether differences between horizons or sites were stronger, we used two-way-ANOVA. To find differences within sites or within horizons, we used one-way ANOVA and Tukey HSD as post-hoc test. We did this for soil parameters, enzyme activities, and fungi:bacteria ratios, as well as for distances between different horizons and within horizons (variability). Before analysis, data were log-transformed or rank-normalized to meet the assumptions for ANOVA. Differences and correlations were assumed to be significant at p < 0.05. Statistics were performed in R 3.0.2 (R Development Core Team, 2013) using the vegan package (Oksanen et al., 2013).

## 3 Results

### 3.1 Enzyme activities

**Fig. 1:**
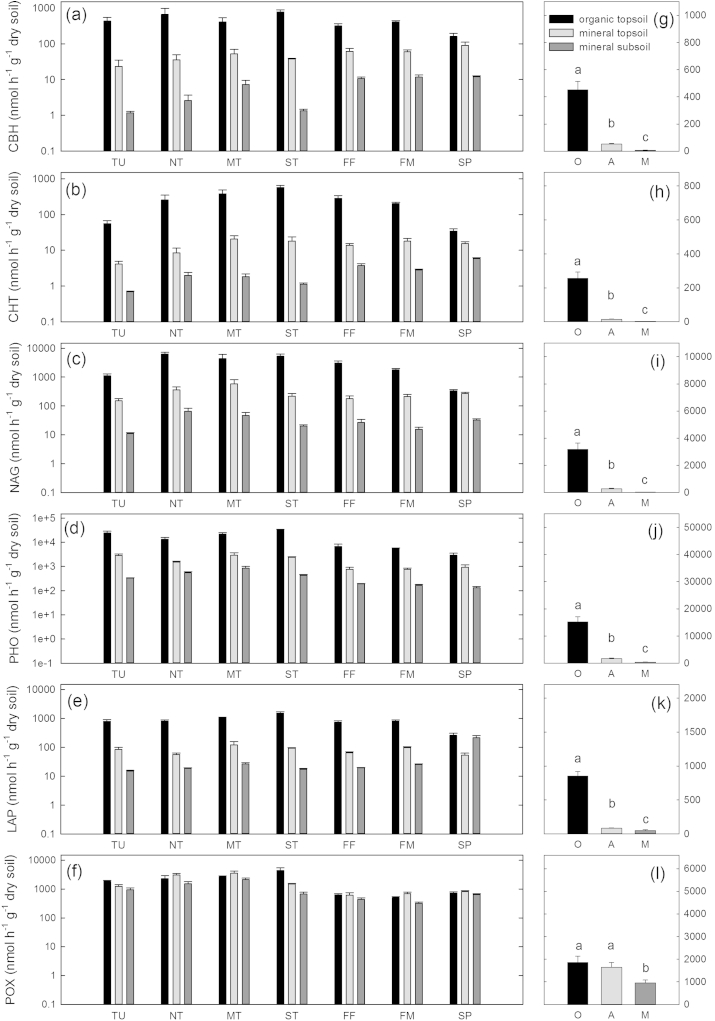
Extracellular enzyme activities per gram dry soil. Left panels (a–f) show activities on a log-scale for each horizon (O are organic topsoils, black; A are mineral topsoils, light gray; M are mineral subsoils, dark gray) at each site (TU = Tundra; NT = northern taiga; MT = middle taiga; ST = southern taiga; FF = forest steppe forest; FM = forest steppe meadow; SP = steppe) individually. Right panels (g–l) show the mean of the individual horizons over all sites. Significant differences for horizon means are derived from ANOVA and Tukey HSD tests and are indicated by small letters (p > 0.05). Results from two-way ANOVAs are given in Table 2.

**Fig. 2:**
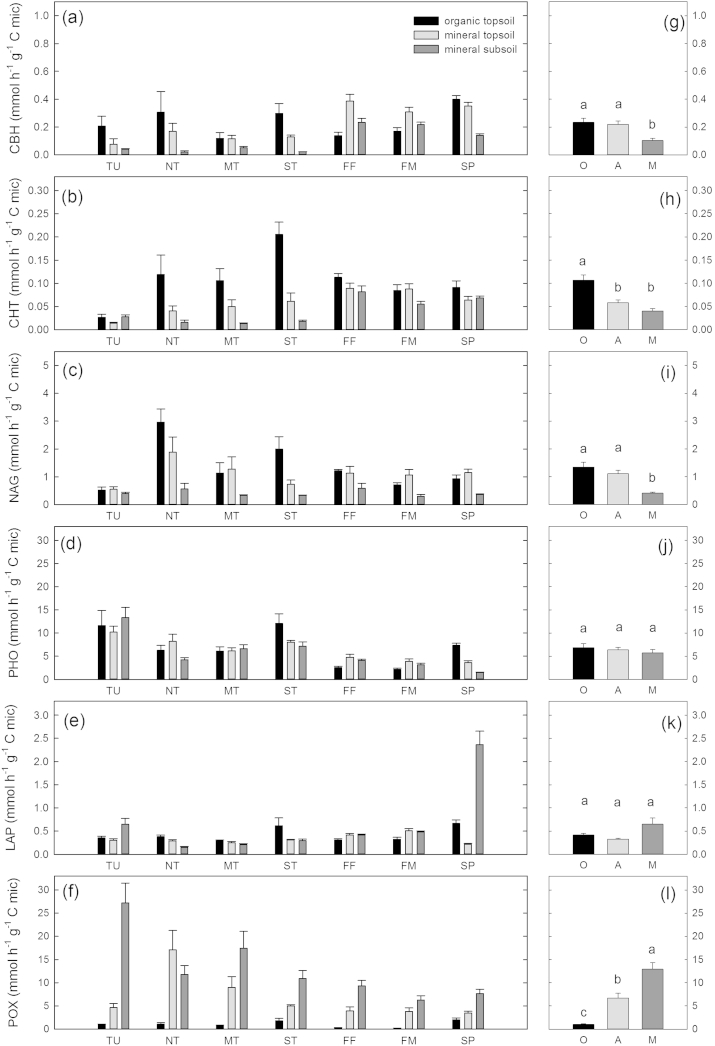
Extracellular enzyme activities per gram microbial C. Left panels (a–f) show activities for each horizon (O are organic topsoils, black; A are mineral topsoils, light gray; M are mineral subsoils, dark gray) at each site (TU = Tundra; NT = northern taiga; MT = middle taiga; ST = southern taiga; FF = forest steppe forest; FM = forest steppe meadow; SP = steppe) individually. Right panels (g–l) show the mean of the individual horizons over all sites. Significant differences for horizon means are derived from ANOVA and Tukey HSD tests and are indicated by small letters (p > 0.05). Results from two-way ANOVAs are given in Table 2.

All measured hydrolytic enzyme activities – calculated per gram dry soil – differed more strongly between horizons than between sites (Table 2). Hydrolytic enzyme activities were highest in organic topsoil horizons followed by mineral topsoils and mineral subsoil horizons, with the exception of LAP in steppe where the highest values were found in organic topsoil horizons and mineral subsoil horizons and lower values were found in mineral topsoil horizons (Fig. 1). The oxidative enzyme POX did not follow this pattern and showed greater differences between sites than between horizons (Table 2, Fig. 1). When hydrolytic enzyme activities were calculated on a microbial C basis, differences between horizons were often smaller than differences between sites (Table 2, Fig. 2). POX activity, on a microbial C basis, was highest in the mineral subsoils followed by mineral topsoils (except northern taiga). The lowest rates were found in organic topsoil horizons at all sites. Differences between sites were ambiguous and POX activities, on a microbial C basis, in mineral subsoil horizons was the only enzyme activity that showed a clear north–south trend and decreased from the tundra site in the north to the steppe site in the South (Fig. 2).

### 3.2 Enzyme patterns

**Fig. 3:**
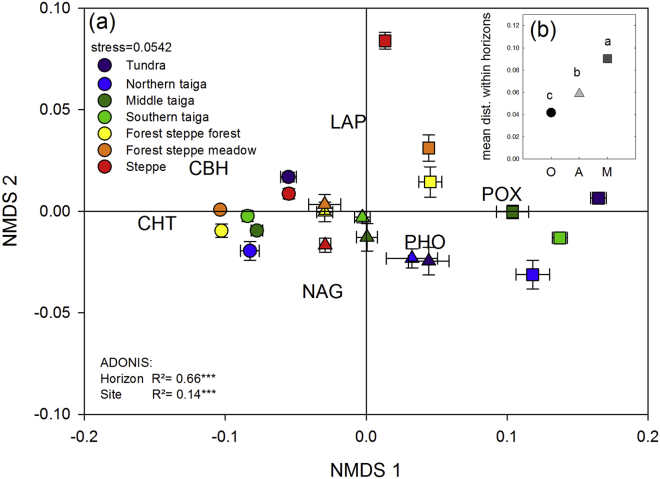
Enzyme patterns. NMDS plot of enzyme patterns calculated from a distance matrix of standardized enzyme activities (a). Symbols are the mean values of the replicated individual horizons of each site. Error bars are SE. Sites are indicated by color (Tundra is purple; northern taiga is blue; middle taiga is dark green; southern taiga light green; forest steppe forest is yellow; forest steppe meadow is orange; steppe is red). Horizons are indicated by different symbols (circles are organic topsoils; triangles are mineral topsoils; squares are mineral subsoils). The insert in the upper right corner (b) shows the distances within the horizons. The results of the ADONIS analysis show that horizon has a stronger effect than site. Asterisks indicate significance (*** mean p > 0.001). (For interpretation of the references to color in this figure legend, the reader is referred to the web version of this article.)

To evaluate differences in the way microbes decompose SOM in different horizons, we used log-transformed and standardized enzyme activities to calculate distance matrixes. The resulting enzyme patterns differed more strongly between horizons than between sites (ADONIS: horizons R^2^ = 0.66, sites R^2^ = 0.14; Fig. 3). The mean distances and thus the variability of enzyme patterns were greatest in the mineral subsoil horizons, followed by mineral topsoil horizons, and organic topsoil horizons (Fig. 3b). Enzyme patterns of different horizons in the South clustered closer together than enzyme patterns of different horizons in the North in the NMDS plot (Fig. 3). This trend was more pronounced for differences (mean distances) between organic topsoils and mineral subsoils, which were significantly higher in the northern sites (tundra and taiga) than in the southern sites (forest-steppe and steppe; Fig. 5a). This trend was weaker but could also be seen for differences between organic topsoils and mineral topsoils (Fig. 5b). The differences between mineral topsoils and mineral subsoils did not show a decrease from North to South (Fig. 5c).

Correlations (Mantel tests) of enzyme patterns with biotic and abiotic factors varied between horizons (Table 3). The most striking difference between organic topsoils and mineral subsoils is the absence of a correlation between enzyme patterns and SOM properties (C, N, C/N ratio) in the mineral subsoils.

### 3.3 Microbial community composition

**Fig. 4:**
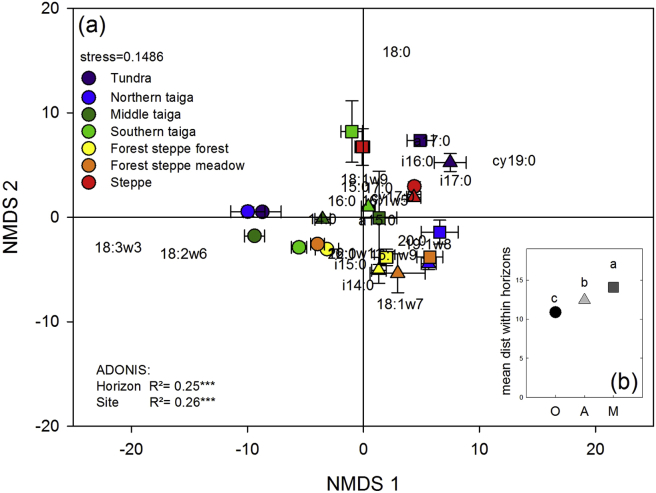
Microbial community composition. NMDS plot of microbial community composition calculated as distance matrix of the relative abundances of all individual PLFA markers (a). Symbols are the mean values of the replicated individual horizons of each site. Error bars are SE. Sites are indicated by color (Tundra is purple; northern taiga is blue; middle taiga is dark green; southern taiga light green; forest steppe forest is yellow; forest steppe meadow is orange; steppe is red). Horizons are indicated by different symbols (circles are organic topsoils; triangles are mineral topsoils; squares are mineral subsoils). The insert in the lower right corner (b) shows the distances within the horizons. The results of the ADONIS analysis show that horizon and site have equal influence. Asterisks indicate significance (*** mean p > 0.001). (For interpretation of the references to color in this figure legend, the reader is referred to the web version of this article.)

**Fig. 5:**
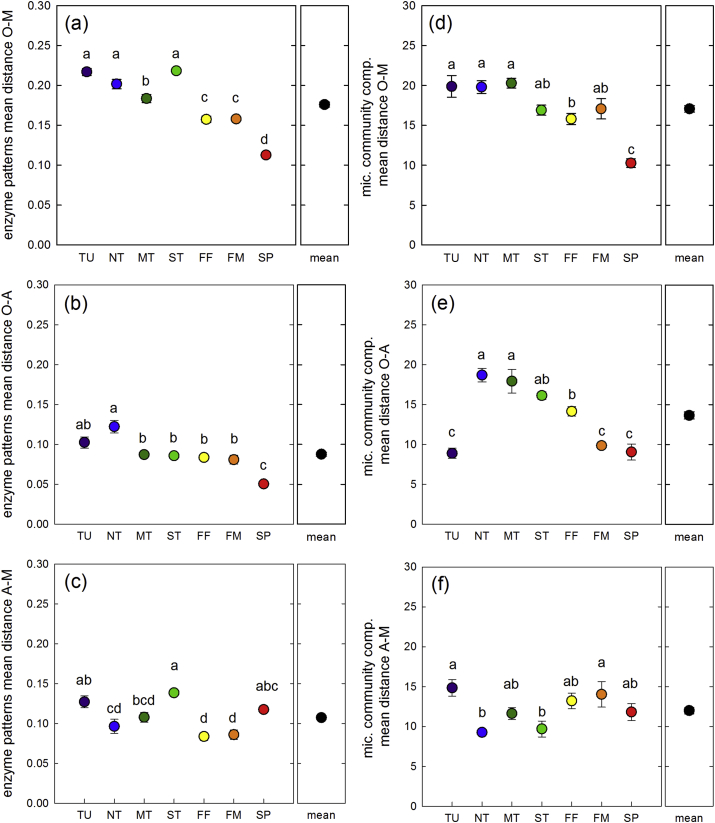
Differences between horizons in enzyme pattern (a–c) and microbial community composition (d–f). Values are the mean distances between the respective shown horizons. Sites are indicated by color (Tundra is purple; northern taiga is blue; middle taiga is dark green; southern taiga light green; forest steppe forest is yellow; forest steppe meadow is orange; steppe is red). Black dots are the mean values over all sites. (For interpretation of the references to color in this figure legend, the reader is referred to the web version of this article.)

**Fig. 6:**
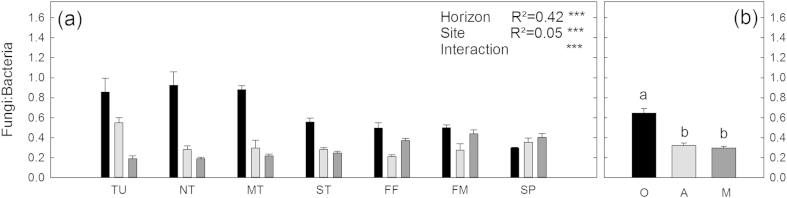
Fungi:bacteria ratios. Left panel (a) shows each horizon (O are organic topsoils, black; A are mineral topsoils, light gray; M are mineral subsoils, dark gray) at each site (TU = Tundra; NT = northern taiga; MT = middle taiga; ST = southern taiga; FF = forest steppe forest; FM = forest steppe meadow; SP = steppe) individually. Right panel (b) shows the mean of the individual horizons over all sites. Results for two-way ANOVA are shown in the left panel. Asterisks indicate significance (*** mean p > 0.001). In the right panel significant differences for horizon means are derived from ANOVA and Tukey HSD tests and are indicated by small letters (p > 0.05).

Microbial community composition differed significantly between horizons and between sites with both factors exerting a similar influence (site R^2^ = 0.26 and horizon R^2^ = 0.25). Differences between horizons were mainly caused by a decrease in fungal markers (18:3ω3 and 18:2ω6) with soil depth (Fig. 4). The differences in microbial community composition between horizons were further reflected in the fungi:bacteria ratios which decreased from organic topsoils, to mineral topsoils and mineral subsoils (Fig. 6). A clear north–south trend is also seen in the fungi:bacteria ratios in the topsoil and therefore differences between horizons were lower in the south located ecosystems. Although differences between horizons were not as pronounced for microbial community composition as for enzyme patterns, the variability within the horizons was also highest in mineral subsoils followed by mineral topsoils and organic topsoils (Fig. 4b). Microbial community composition in organic topsoils and mineral subsoils differed more in the North than in the South (Fig. 5d). With the exception of tundra, differences between organic topsoils and mineral topsoils also showed a decrease from North to South (Fig. 5e). Correlations of microbial community composition with biotic and abiotic factors were similar to those observed for enzyme patterns (Table 3). The correlations of microbial community composition and SOM parameters (C, N, CN ratio, δ^13^C) were strongest in the organic topsoils, but decreased to mineral topsoils and further to mineral subsoils (Table 3).

## 4 Discussion

### 4.1 Enzyme activities and enzyme patterns

Subsoil horizons differ in a range of physical and chemical parameters from topsoil horizons (Fierer et al., 2003; Salome et al., 2010; Rumpel and Kögel-Knabner, 2011). They also exhibit a reduced influence of plants and a higher proportion of the present SOM is associated with minerals (Rumpel and Kögel-Knabner, 2011). These factors are most likely responsible for the clear separation of enzyme patterns according to horizons (R^2^ = 0.66), which was stronger than the differences in enzyme patterns between sites (R^2^ = 0.14; Fig. 3), in our study. A similar picture in enzyme patterns, with R^2^ for horizon of 0.48 and R^2^ for site of 0.23 could be found when POX was not included in the analysis (data not shown). The higher R^2^ for horizon when POX was included, indicate that the different change of oxidative and hydrolytic enzyme activities with depth was a major factor responsible for the distinct enzyme patterns in topsoils and subsoils. While hydrolytic enzyme activities generally decreased with depth, POX activities did not change from topsoil to subsoil (Fig. 1). An explanation for this might be that the activity of hydrolytic enzymes is often directly related to SOM content (Sinsabaugh et al., 2008; Schnecker et al., 2014). Since SOM content and the amount of regular polymers, that can be broken down hydrolytically, decrease with depth (Rumpel and Kögel-Knabner, 2011), hydrolytic enzyme activities can be expected to decrease accordingly. Oxidative enzymes, in contrast, are unspecific and are often not produced to directly acquire nutrients (Sinsabaugh, 2010). Instead, oxidative enzymes can degrade humic complexes and thereby free substrates for other enzymes (Hobbie and Horton, 2007; Talbot et al., 2008) or degrade toxic substances such as phenols (Sinsabaugh, 2010). The production of oxidative enzymes might thus be related to the amount of irregular polymers or the amount of toxins, which are both independent from SOM content. In addition, and in contrast to hydrolytic enzymes, oxidative enzymes are preferentially stabilized on mineral surfaces and might thus prevail longer in mineral subsoils (Kramer et al., 2013). Overall, the contrasting behavior of hydrolytic and oxidative enzymes presumably led to the more pronounced differences in enzyme pattern between horizons than between sites.

Differences between sites and a latitudinal trend could be found for differences between enzyme patterns (expressed as mean distances), in organic topsoil and mineral subsoil horizons (Figs. 3 and 5). We found that enzyme patterns in topsoils and subsoils were most similar at the southernmost site, although organic topsoils and mineral subsoils in this steppe ecosystem were up to one meter apart from each other (Table 1). In contrast, at the tundra site enzyme patterns showed the greatest differences of all sites between organic topsoils and mineral subsoils, which are less than 50 cm apart. This trend from North to South was not caused by the variability of enzyme patterns in topsoils, but by the large variability in mineral subsoils (Fig. 3). This is in contrast to our hypothesis that greater differences between ecosystems would occur in organic topsoil horizons due to the diverse litter inputs in different ecosystems. Again, these differences between ecosystems in mineral horizons could be found with and without POX, but POX activity, on a microbial C basis (Fig. 2) was the only individual enzyme activity that decreased from North to South. Also in this case enzyme activities might have been controlled by physical parameters, which vary especially in subsoils of different ecosystems. Anoxia and water saturation for instance are common features of subsoil horizons of high latitude ecosystems, whereas they can be neglected as important factors in arid steppe subsoils. Fluctuating oxygen conditions might lead to abiotic oxidation of organic material in the presence of iron or manganese and mimic oxidative enzyme activities (Bach et al., 2013; Hall and Silver, 2013). This might explain the high oxidative activities found in the northern ecosystems. Lack of oxygen influences oxidative enzyme gravely and although it has been proposed that anoxia does not directly affect hydrolytic enzyme activities (Hall et al., 2014), individual enzymes or their substrates might be differently stabilized on mineral surfaces (Turner et al., 2014). These stabilization mechanisms can be influenced by physical and chemical factors such as O_2_ availability and pH or by the mineralogical composition of soils (von Lützow et al., 2006). Along the transect parent material changed from marine deposits at the tundra site to fluvi-glacial deposits in the northern and middle taiga to eolian deposits south of the middle taiga (Archipov et al., 1970). These differences in parent material might be reflected in the mineralogical composition of the soils at these sites.

### 4.2 Enzyme patterns and microbial community composition

Physical factors in subsoil horizons might also have indirectly influenced enzyme patterns by affecting microbial community composition (Schnecker et al., 2014). In all three soil horizon types, we found significant correlations between enzyme patterns and microbial community composition (Table 3). Microbial community composition itself was significantly different between horizons along this 1500 km long Siberian transect (ADONIS in Fig. 4). The differences between horizons even outranked differences between ecosystems in topsoil horizons, with a mean distance between organic topsoils and mineral subsoils of 17.1 ± 0.4 (Fig. 5) and mean distance within organic topsoil horizons of 10.9 ± 0.2 (Fig. 4b). Similar trends were found for North-American forest systems where the variability of the microbial community composition was greater within soil profiles than between 54 topsoil horizons collected from a wide range of ecosystems (Eilers et al., 2012). This clear picture of the stronger influence of depth than of geographical distance on microbial community composition becomes however blurred when subsoil horizons were also considered. When we included mineral topsoils and mineral subsoils in the statistical analyses, we found significant differences in microbial community composition between ecosystems (R^2^ = 0.26), which were as strong as differences between horizons (R^2^ = 0.25).

These findings might have two implications: First, the consistent correlations of enzyme patterns and microbial community composition, and the different enzyme activities on a microbial C basis (Fig. 2), point to distinct functional capacities of individual microbial communities in different soil horizons. This also indicates that enzyme patterns are an estimate for a functional community composition. Second, the differences between horizons were more pronounced in enzyme patterns than in the microbial community composition (Fig. 5), which might indicate an additional physiological adaptation of the microbial communities on top of community shifts from topsoils to subsoils.

### 4.3 Enzyme patterns and soil organic matter

So far, we have shown relations of enzyme patterns and microbial community composition and argued the potential controls of physical parameters over both. Enzyme patterns may however also reflect the availability of different substrates, as well as microbial energy and nutrient demand, and are often related to C/N ratios of the microbial biomass or of the SOM (Sinsabaugh et al., 2008). While, in this present study, enzyme patterns and microbial community composition were related to C and N content in the organic topsoil horizons, these relations were not found in mineral topsoils and mineral subsoils. In contrast to SOM quantity, SOM chemistry might be more important in subsoil horizons. Here, the distinct microbial communities that are presumably adapted to the different environments along the transect could have led to a diverging chemical composition of SOM, similar to a proposed diverging of litter chemistry with ongoing decomposition (Wickings et al., 2012). This divergence has been found in a litter decomposition study and has not been shown for SOM. Enzyme patterns might, nonetheless, reflect a diverged SOM chemistry, and therefore show greater variability in subsoils, where a high proportion of SOM is microbially transformed, than in topsoils, where a higher amount of plant components is present.

In summary our findings show that topsoil horizons and subsoil horizons harbor different microbial communities, which support distinct ways to decompose the available SOM. In accordance to our first hypothesis (i.e., that enzyme patterns in topsoil and subsoil horizons are both related to the same key parameters), we found that differences in enzyme patterns between horizons outranked the differences between ecosystems. However, in contradiction to our third hypothesis (i.e., that enzyme patterns would be more variable and show greater differences between ecosystems in topsoil than in subsoil horizons), we found a higher variability of enzyme patterns in subsoil horizons, which might have been caused by an interplay of physical conditions, microbial community composition and chemical composition of SOM. Although we were not able to identify and describe the mechanisms that shape the microbial community and control enzyme patterns in subsoil horizons in detail, we found that enzyme patterns and thus the strategy of the microbial community to decompose SOM, were not related to SOM content and C/N ratios, which contradicts our second hypothesis. In subsoil horizons, the microbial community, with its functional abilities, might be responsible for the way in which SOM is decomposed. In addition to SOM content or C/N ratios, the microbial community composition might therefore constitute an important factor controlling decomposition rates, especially in subsoil horizons.

## Figures and Tables

**Table 1:** Basic soil and site characterization of sites along the latitudinal transect. MAT is mean annual temperature; MAP is mean annual precipitation. Aridity index has a threshold for drylands at 0.65 (Maestre et al., 2012).

	Coordinates	MAT °C	MAP mm	Aridity index	Soil type	Organic topsoils		Mineral topsoils		Mineral subsoils	
						Horizon	Depth cm	Horizon	Depth cm	Horizon	Depth cm
Tundra	67°16′N 78°50′E	−7.6	391	1.30	Turbic Cryosol	O	0–6	A	2–13	Bg, BCg	6–57
Northern taiga	63°17′N 74°32′E	−4.6	430	1.06	Histic Podzol	Oi, Oe	0–22	AE, EA	8–30	Bg	14–47
Middle taiga	60°09′N 71°43′E	−2.2	438	0.89	Endogleyic Regosol	Oi	0–6	A, AE, EA	6–14	E, EA	12–55
Southern taiga	58°18′N 68°35′E	−0.5	396	0.71	Albic Podzol	Oi	0–7	A, AE	4–18	E, EA	15–59
Forest steppe: Forest	56°14′N 70°43′E	0.7	340	0.53	Haplic Phaeozem	O, Oa	0–10	A	7–46	B	57–109
Forest steppe: Meadow	56°14′N 70°43′E	0.7	340	0.53	Luvic Phaeozem	Oa	0–7	A	4–35	Bt	26–84
Steppe	54°41′N 71°38′E	1.0	309	0.44	Calcic Kastanozem	OA	0–12	Ak	8–37	Bk	27–109

**Table 2:** Two-way ANOVA R^2^ for enzyme activities, based on dry soil and on microbial C basis; only significant differences are shown. Bold letters indicate whether horizon or site have the stronger influence on enzyme activities (higher R^2^). Asterisks indicate significance (**mean p < 0.01; ***mean p < 0.001).

	Activities per g DM			Activities per g Cmic		
	Horizon	Site	Interaction	Horizon	Site	Interaction
cellobiohydrolase (CBH)	**0.80**	0.05	***	0.36	**0.52**	***
chititriosidase (CHT)	**0.82**	0.06	***	0.24	**0.32**	***
N-acetyl-glucosaminidase (NAG)	**0.84**	0.06	***	**0.43**	0.16	**
phosphatase (PHO)	**0.79**	0.16	***	0.03	**0.56**	***
leucine-amino-peptidase (LAP)	**0.83**	0.01	***	0.05	**0.28**	***
phenoloxidase (POX)	0.03	**0.17**	***	**0.73**	0.10	***

**Table 3:** Results of Mantel tests of enzyme patterns (distance matrix with standardized enzyme activities) and microbial community composition (distance matrix of relative amounts of PLFA) with abiotic and biotic variables. Only significant relations are shown (p < 0.05). Values are R.

	Enzyme patterns			Microbial community composition		
	Organic topsoils	Mineral topsoils	Mineral subsoils	Organic topsoils	Mineral topsoils	Mineral subsoils
C content	0.06			0.29	0.03	
N content	0.22			0.30		
SOM C/N	0.05	0.04		0.27	0.04	
SOM δ^13^C	0.27			0.29	0.12	0.03
Microbial C				0.11	0.03	
Microbial N	0.03		0.02	0.18		
Microbial C/N		0.05		0.24	0.02	
pH	0.02	0.03	0.29	0.05	0.14	0.09
Water holding capacity	0.03		0.20	0.25	0.05	
Fungi:bacteria ratio	0.10	0.03	0.24	0.49	0.08	0.20
Mic. community comp.	0.16	0.04	0.17	–	–	–
Enzyme patterns	–	–	–	0.16	0.04	0.17
