# Supplementary material for: Microbial community composition shapes enzyme patterns in topsoil and subsoil horizons along a latitudinal transect in Western Siberia
Source: Soil Biol Biochem. 2015 Apr;83:106–15. doi: 10.1016/j.soilbio.2015.01.016 (PMC4381299; doi:10.1016/j.soilbio.2015.01.016)
Supplement: Supplementary file 1 [file mmc1.docx]

Table S1 Soil organic matter properties, microbial biomass properties and soil parameters. Values for horizons at individual sites are mean values of the 5 replicated soil pits (mean ± standard error). Values for overall means are given as mean and the coefficient of variance in parentheses

|  | C content | N content | SOM C/N | SOM δ^13^C | Microbial C | Microbial N | Microbial C/N | pH | WHC |
| --- | --- | --- | --- | --- | --- | --- | --- | --- | --- |
|  | mg g^-1^ dry soil | mg g^-1^ dry soil |  | ‰ | µg g^-1^ dry soil | µg g^-1^ dry soil |  |  | g H_2_O g^-1^ dry soil |
| Tundra |  |  |  |  |  |  |  |  |  |
| Organic topsoils | 308 ± 33.4 | 8.81 ± 0.59 | 34.9 ± 3.09 | -26.6 ± 0.16 | 2289 ± 326 | 328 ± 36.1 | 6.89 ± 0.30 | 3.78 ± 0.08 | 4.70 ± 0.61 |
| Mineral topsoils | 30.4 ± 2.73 | 1.83 ± 0.11 | 16.4 ± 0.66 | -25.7 ± 0.04 | 290 ± 48.7 | 30.5 ± 4.89 | 9.54 ± 0.28 | 3.70 ± 0.03 | 0.79 ± 0.08 |
| Mineral subsoils | 4.51 ± 0.39 | 0.39 ± 0.03 | 11.7 ± 0.27 | -24.7 ± 0.07 | 31.6 ± 6.23 | 1.89 ± 0.24 | 19.2 ± 3.69 | 3.83 ± 0.04 | 0.25 ± 0.01 |
| Northern taiga |  |  |  |  |  |  |  |  |  |
| Organic topsoils | 448 ± 6.25 | 12.5 ± 0.24 | 35.9 ± 0.64 | -28.3 ± 0.13 | 2133 ± 46.7 | 332 ± 11.4 | 6.46 ± 0.21 | 2.76 ± 0.04 | 6.61 ± 0.30 |
| Mineral topsoils | 37.0 ± 2.79 | 1.36 ± 0.07 | 27.4 ± 1.78 | -27.1 ± 0.24 | 201 ± 23.6 | 13.7 ± 1.50 | 14.8 ± 1.18 | 3.06 ± 0.05 | 0.71 ± 0.02 |
| Mineral subsoils | 8.17 ± 1.53 | 0.50 ± 0.05 | 15.7 ± 1.37 | -25.9 ± 0.16 | 133 ± 13.7 | 3.43 ± 0.27 | 38.7 ± 2.41 | 3.72 ± 0.05 | 0.41 ± 0.03 |
| Middle taiga |  |  |  |  |  |  |  |  |  |
| Organic topsoils | 426 ± 21.9 | 17.4 ± 0.91 | 24.5 ± 0.48 | -29.0 ± 0.16 | 3669 ± 342 | 505 ± 51.5 | 7.33 ± 0.34 | 3.66 ± 0.05 | 4.99 ± 0.59 |
| Mineral topsoils | 74.7 ± 15.4 | 3.46 ± 0.58 | 20.8 ± 1.65 | -26.9 ± 0.21 | 489 ± 104 | 47.4 ± 11.7 | 11.0 ± 0.79 | 3.32 ± 0.07 | 0.97 ± 0.10 |
| Mineral subsoils | 16.7 ± 3.36 | 0.97 ± 0.12 | 16.3 ± 1.53 | -26.5 ± 0.23 | 136 ± 24.5 | 5.43 ± 0.77 | 25.2 ± 2.46 | 3.48 ± 0.04 | 0.65 ± 0.02 |
| Southern taiga |  |  |  |  |  |  |  |  |  |
| Organic topsoils | 398 ± 16.4 | 15.8 ± 0.79 | 25.4 ± 0.71 | -28.6 ± 0.21 | 3065 ± 583 | 628 ± 71.0 | 4.82 ± 0.61 | 4.26 ± 0.09 | 5.85 ± 0.83 |
| Mineral topsoils | 43.4 ± 3.26 | 3.11 ± 0.16 | 14.0 ± 0.72 | -26.8 ± 0.03 | 302 ± 19.6 | 36.3 ± 2.91 | 8.42 ± 0.50 | 3.62 ± 0.07 | 0.76 ± 0.02 |
| Mineral subsoils | 4.56 ± 0.22 | 0.49 ± 0.03 | 9.40 ± 0.20 | -25.3 ± 0.12 | 61.4 ± 5.36 | 3.32 ± 0.14 | 18.4 ± 0.95 | 3.83 ± 0.04 | 0.30 ± 0.02 |
| Forest steppe: Forest |  |  |  |  |  |  |  |  |  |
| Organic topsoils | 293 ± 21.5 | 17.7 ± 1.12 | 16.5 ± 0.27 | -27.9 ± 0.10 | 2504 ± 381 | 399 ± 59.6 | 6.31 ± 0.39 | 6.64 ± 0.33 | 3.83 ± 0.41 |
| Mineral topsoils | 45.6 ± 4.04 | 3.57 ± 0.39 | 12.9 ± 0.22 | -25.7 ± 0.21 | 156 ± 8.43 | 11.5 ± 0.71 | 13.6 ± 0.28 | 4.26 ± 0.05 | 0.78 ± 0.03 |
| Mineral subsoils | 5.16 ± 0.13 | 0.52 ± 0.03 | 10.1 ± 0.31 | -25.4 ± 0.14 | 46.9 ± 1.72 | 2.9 ± 0.11 | 16.3 ± 0.67 | 4.06 ± 0.04 | 0.62 ± 0.01 |
| Forest steppe: Meadow |  |  |  |  |  |  |  |  |  |
| Organic topsoils | 202 ± 20.3 | 14.0 ± 1.40 | 14.4 ± 0.14 | -27.6 ± 0.06 | 2585 ± 330 | 390 ± 27.2 | 6.53 ± 0.42 | 5.54 ± 0.23 | 3.68 ± 0.18 |
| Mineral topsoils | 25.0 ± 1.65 | 1.90 ± 0.12 | 13.1 ± 0.10 | -26.2 ± 0.08 | 189 ± 20.0 | 14.0 ± 1.52 | 13.5 ± 0.42 | 4.15 ± 0.03 | 0.66 ± 0.03 |
| Mineral subsoils | 5.85 ± 0.31 | 0.55 ± 0.03 | 10.7 ± 0.19 | -25.9 ± 0.05 | 53.2 ± 3.61 | 2.72 ± 0.16 | 19.6 ± 0.91 | 4.02 ± 0.07 | 0.61 ± 0.01 |
| Steppe |  |  |  |  |  |  |  |  |  |
| Organic topsoils | 36.9 ± 2.65 | 3.33 ± 0.22 | 11.1 ± 0.12 | -25.5 ± 0.11 | 401 ± 65.3 | 36.1 ± 6.58 | 11.3 ± 0.39 | 4.62 ± 0.09 | 0.72 ± 0.02 |
| Mineral topsoils | 20.1 ± 2.44 | 1.84 ± 0.19 | 10.8 ± 0.23 | -25.1 ± 0.06 | 247 ± 34.0 | 17.9 ± 2.33 | 13.9 ± 0.50 | 5.08 ± 0.28 | 0.57 ± 0.01 |
| Mineral subsoils | 7.16 ± 0.73 | 0.79 ± 0.09 | 9.15 ± 0.16 | -24.9 ± 0.07 | 87.9 ± 6.37 | 5.00 ± 0.72 | 19.5 ± 2.70 | 7.92 ± 0.36 | 0.51 ± 0.02 |
| Mean (CV) |  |  |  |  |  |  |  |  |  |
| Organic topsoils | 302 (0.47) | 12.8 (0.40) | 23.2 (0.41) | -27.6 (0.04) | 2378 (0.51) | 374 (0.52) | 7.09 (0.29) | 4.47 (0.28) | 4.34 (0.48) |
| Mineral topsoils | 39.9 (0.56) | 2.45 (0.44) | 16.6 (0.35) | -26.2 (0.03) | 270 (0.55) | 24.8 (0.69) | 12.1 (0.22) | 3.88 (0.18) | 0.75 (0.22) |
| Mineral subsoils | 7.62 (0.68) | 0.61 (0.39) | 11.9 (0.28) | -25.5 (0.03) | 80.5 (0.58) | 3.64 (0.42) | 22.7 (0.38) | 4.44 (0.34) | 0.49 (0.30) |
